# Lymphomatosis cerebri caused by adult T cell leukemia/lymphoma: a differential diagnosis for depression: a case report

**DOI:** 10.1186/s13256-024-04666-1

**Published:** 2024-07-31

**Authors:** Satoshi Inaba, Masataka Kudo, Hironori Kamano, Yoshihiro Ohishi, Junichi Kiyasu, Takashi Watari

**Affiliations:** 1 Department of General Internal Medicine, Iizuka Hospital, 3-83 Yoshino-Machi, Iizuka, Fukuoka 820-8505 Japan; 2 Department of General Internal Medicine, Fukuchiyama City Hospital, Kyoto, Japan; 3 Department of Clinical Epidemiology, Kochi Medical School, Nankoku, Japan; 4 Department of Radiology, Iizuka Hospital, Fukuoka, Japan; 5 Department of Diagnostic Pathology, Iizuka Hospital, Fukuoka, Japan; 6 Department of Hematology, Iizuka Hospital, Fukuoka, Japan; 7 General Medicine Center, Shimane University Hospital, Shimane, Japan; 8 Department of Medicine, University of Michigan Medical School, Ann Arbor, MI USA

**Keywords:** Adult T cell leukemia/lymphoma, Lymphomatosis cerebri, Central nervous system neoplasms, Nervous system diseases, Lymphoma, Autopsy, Depression

## Abstract

**Background:**

Primary central nervous system lymphoma is rare, and primary central nervous system T cell lymphoma is relatively uncommon, contributing to < 5% of all cases. Lymphomatosis cerebri, a rare subtype of primary central nervous system lymphoma, is characterized by extensive white-matter lesions on magnetic resonance imaging and nonspecific symptoms, such as cognitive decline and depression. Reports of lymphomatosis cerebri in adult T cell leukemia/lymphoma are limited.

**Case presentation:**

A 49-year-old Japanese man gradually developed insomnia, anorexia, and weight loss over a 2-month period following work-related promotion. Initially diagnosed with depression, his condition rapidly deteriorated with cognitive decline and motor dysfunction. Despite various treatments, his symptoms persisted within a month. Upon admission, the presence of neurological abnormalities suggestive of a central nervous system disorder raised suspicion of a cerebral lesion. Diagnostic tests revealed extensive brain lesions on imaging and the presence of atypical lymphocytes (flower cells) in the cerebrospinal fluid. The patient was diagnosed with lymphomatosis cerebri due to adult T cell leukemia/lymphoma, a rare presentation in the literature. Due to irreversible brainstem damage and poor neurological prognosis, aggressive treatment was not initiated, and the patient died, with an autopsy confirming the diagnosis.

**Conclusion:**

Lymphomatosis cerebri with adult T cell leukemia/lymphoma is very rare. It is crucial to promptly consider lymphomatosis cerebri as a differential diagnosis, particularly in cases of rapid cognitive decline and poor treatment response. Recognition of lymphomatosis cerebri as an important differential diagnosis for cognitive decline, and depression is necessary for timely intervention and management. Further research is required to better understand this unique and rare presentation in adult T cell leukemia/lymphoma.

## Background

Primary central nervous system (CNS) lymphoma is a rare condition, accounting for 0.45 per 100,000 population [1]. Diffuse large B cell lymphoma accounts for most primary CNS lymphomas, whereas primary CNS T cell lymphoma is relatively uncommon, accounting for 2% of all primary CNS lymphomas [2]. Primary CNS lymphomas are generally characterized by clinical and neuroimaging findings consistent with those of single or multiple intracranial mass lesions. However, lymphomatosis cerebri (LC) is a rare subtype of primary CNS lymphoma, presenting with extensive white-matter lesions on magnetic resonance imaging (MRI) [3] and nonspecific symptoms, such as cognitive decline and depression [4, 5]. Given the nonspecific clinical and neuroimaging characteristics, the diagnosis of LC often presents a considerable challenge, requiring careful consideration of various potential causes. CNS infections, inflammatory conditions, toxic insults, and metabolic disorders can imitate the radiological features observed in LC [5, 6], leading to a delayed and incorrect diagnosis. The median overall survival in untreated patients was 1.97 months, while in patients receiving methotrexate-based therapies, it was 13.8 months. Particularly, given that the initiation of therapeutic measures during a favorable Karnofsky performance status is positively correlated with a favorable prognosis, early diagnosis and prompt intervention are deemed pivotal [4]. In previous LC reports, the proportion of T cell type is 8.9%, and none of them was caused by human T cell leukemia virus type 1 (HTLV-1) [7–10]. Herein, we present the case of LC associated with adult T cell leukemia/lymphoma, aiming to contribute toward expediting the diagnostic process in such scenarios.

## Case presentation

A 49-year-old Japanese man with no history of physical or mental illness gradually developed symptoms of insomnia, anorexia, and weight loss approximately 1 month after being promoted at work and after an offer to be transferred at work 3 months before visiting our hospital. One month prior to his visit, he consulted an internist with complaints of poor concentration and somnolence during the day and was suspected of having depression. He was subsequently referred to a psychiatrist who prescribed duloxetine 20 mg/day. However, his spontaneity rapidly declined, and he required assistance with toileting and walking. The following week, he had difficulty talking, was judged to be comatose, and was subsequently admitted to a psychiatric hospital. At the time of admission, the patient could barely transfer himself to a wheelchair or open his eyes, and he displayed poor voluntary body movements and eye pursuit. He rapidly became bedridden. Intranasal injections of vortioxetine (a selective serotonin reuptake inhibitor) 10 mg/day, aripiprazole 3 mg/day, lorazepam 2 mg/day, and mirtazapine 15 mg/day were administered; however, the patient’s condition persisted with a Japan Coma Scale score of 200 points (equivalent to approximately six points on the Glasgow Coma Scale score [11]). He was suspected of having malignant catatonia, and despite being treated with intramuscular injections of lorazepam 2 mg/day and diazepam 20 mg/day, his symptoms did not improve. After 3 weeks of psychiatric inpatient treatment, he was referred to our hospital to exclude organic disease.

At the time of admission, the patient had a Glasgow Coma Scale score of five points (E1V1M3) and presented the following vital signs: blood pressure, 153/106 mmHg; pulse rate, 106 beats per minute; respiratory rate, 21 breaths per minute; and oxygen saturation, 94% on room air. The general physical examination findings were normal. The neurological examination revealed the pupil size to be 3/3 mm, and light and eyelash reflexes to be absent bilaterally. The ocular position was predominantly left, and leftward horizontal nystagmus was observed in both eyes. Muscle tone was strong in the extremities, and tendon reflexes were enhanced, with right predominance in the upper body and left predominance in the lower body: brachioradialis^+/+^, biceps^++^/^+^, triceps^++/+ ^, patellar^+/++^, Achilles^+/+^, ankle clonus^−/+^, Babinski, Chaddock, Hoffman, and Trömner reflexes were absent, and there was a slight escape response to pain stimuli in all extremities.

**Fig. 1:**
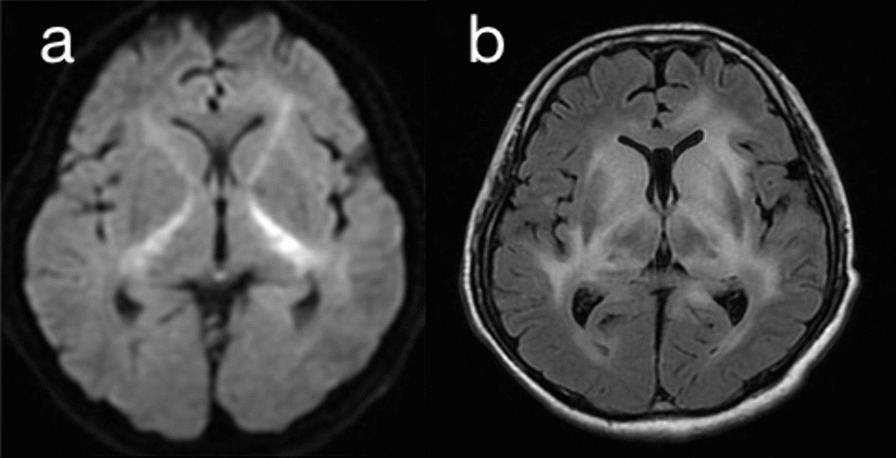
Head magnetic resonance imaging on admission day. **a** With diffusion-weighted imaging. **b** With fluid-attenuated inversion recovery imaging

**Fig. 2:**
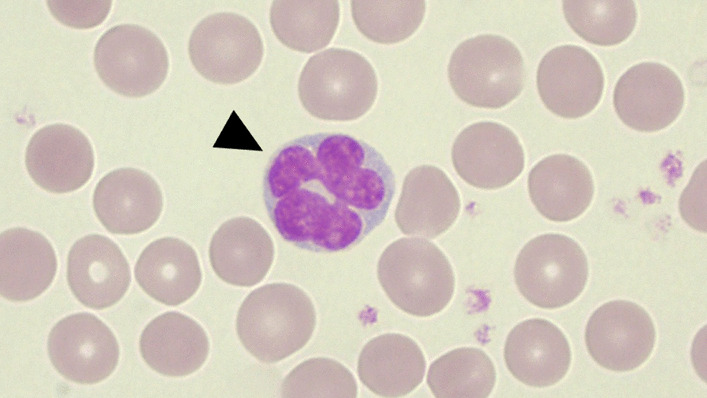
Flower cells (arrow) from the patient’s peripheral blood using a 100× objective lens

**Fig. 3:**
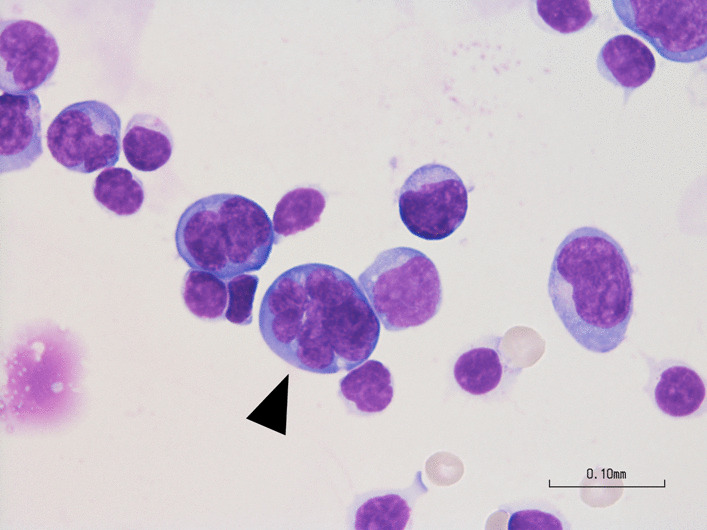
Flower cells (arrow) from the patient’s cerebrospinal fluid using a 100× objective lens

Blood tests revealed a white blood cell count of 17,420/μL, with differential percentages as follows: neutrophils 86.6%, lymphocytes 7.3%, monocytes 3.4%, eosinophils 1.2%, and basophils 0.6%. Red blood cell parameters (hemoglobin 15.2 g/dL, hematocrit 50.5%) and platelet count (297,000/μL) were within normal ranges. Biochemical and immunological tests showed a C-reactive protein level of 1.67 mg/L, with other data within normal limits. The syphilis, antinuclear antibody, copper, and vitamin B1 test results were also normal. Computed tomography (CT) of the head revealed symmetrical extensive hypoabsorption areas in the brain parenchyma. Head MRI showed marked diffusion abnormalities in the bilateral entorhinal to cerebral peduncles on diffusion-weighted imaging (DWI) (Fig. 1a). In addition, the extensive cerebral high-intensity lesion was observed on fluid-attenuated inversion recovery (FLAIR) imaging (Fig. 1b). A mildly elevated cerebrospinal fluid (CSF) cell count of 70/mm^3^, with a protein level of 52 mg/dL and glucose level of 56 mg/dL, was observed, suggesting encephalitis, neurosyphilis, drug-induced encephalopathy, CNS lupus, and malignant lymphoma. Electroencephalography mostly revealed generalized 1–2 Hz high-amplitude delta waves, with no apparent epileptic waves. On the third day of hospitalization, a May–Giemsa stain was performed on the peripheral blood, revealing the presence of flower cells (Fig. 2). Subsequently, a cytopathological examination of CSF also revealed flower cells (Fig. 3). Flow cytometric analysis of CSF showed a predominance of T cells. The immunophenotypic profile indicated that 82.6% of the cells were CD2^+^, 76.3% were CD3^+^, 73.5% were CD5^+^, and 74.7% were CD7^+^. The analysis also revealed the presence of both CD4 and CD8 T cells, with 42.4% and 37.2% positivity, respectively, resulting in a CD4/CD8 ratio of 1.1. These findings align with the expected T cell predominance in cases of adult T cell leukemia/lymphoma. The patient was also positive for HTLV-1 antibody; therefore, we suspected HTLV-1-related primary CNS lymphoma. The route of HTLV-1 infection remained unknown as the patient’s mother had no history of testing, there were no records of blood transfusions, and both the patient’s sexual history and history of substance abuse were inaccessible due to communication barriers. In this patient, the human immunodeficiency virus antibody test was negative. In random skin biopsies, no clustering of atypical lymphocytes within blood vessels was observed. In bone marrow aspiration and trephine biopsy, normocellular marrow was noted, with approximately 10% of small lymphocytes being CD3^+^/CD20^−^, although atypia was limited.

**Fig. 4:**
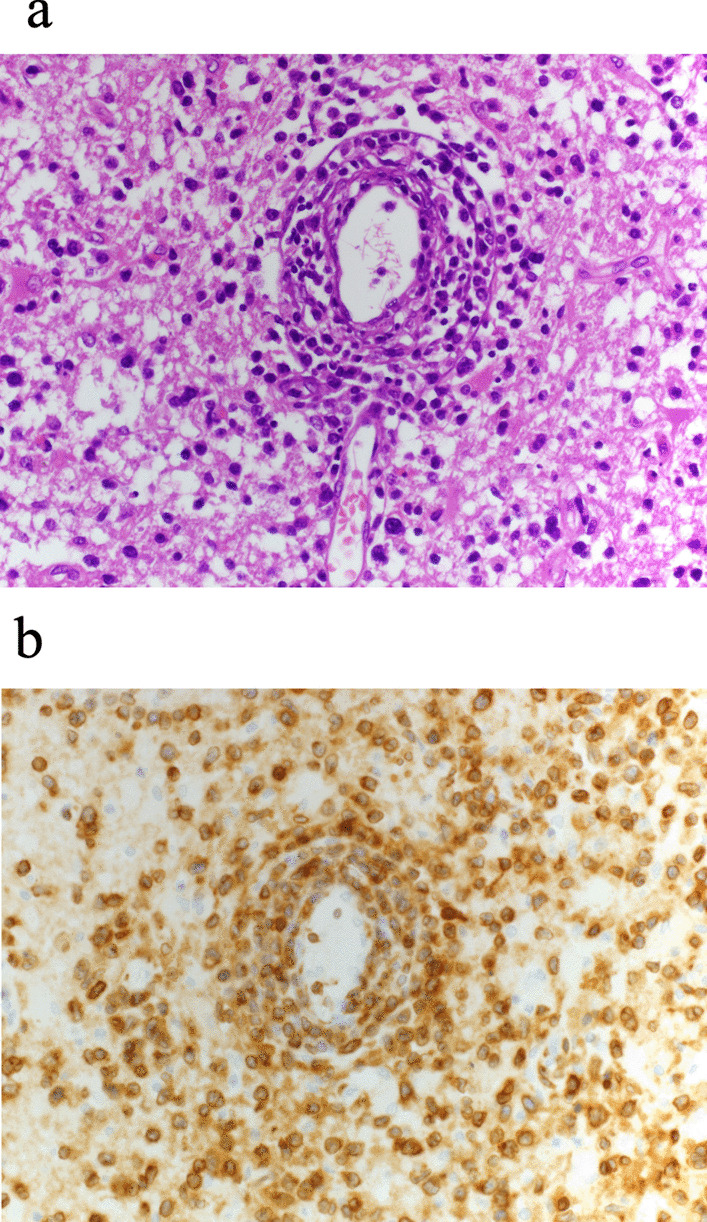
Atypical T cell lymphocyte-like cells identified entirely in the patient’s brain tissue from the pathological autopsy using a 400× objective lens. **a** Hematoxylin and eosin stain demonstrating diffuse infiltration of atypical lymphocyte-like cells. **b** Immunohistochemical staining for CD3 in brain tissue indicating positive staining in lymphocyte-like cells

The clinical diagnosis of HTLV-1-associated primary CNS lymphoma was established based on high-level positivity for HTLV-1 antibody (≥ 8192 times), positive spinal fluid cytology (flower cells), and absence of any apparent primary disease on bone marrow and skin biopsies and systemic contrast-enhanced CT [12]. After consulting with a hematologist, we decided not to perform a brain biopsy or initiate aggressive treatment, including chemotherapy, as irreversible damage to the brainstem was suspected [13, 14], and the neurological prognosis was deemed poor. The patient received palliative management while undergoing hydration via peripheral intravenous fluids. Although his consciousness was not restored, with the intervention of a palliative care team, including grief support for the family, he remained in a peaceful state accompanied by his family. Six weeks after admission to our hospital, the patient peacefully passed away. The pathological autopsy showed diffuse infiltration of T cell atypical lymphocyte-like cells into the brain tissue (Fig. 4a), and immunohistochemical analysis revealed that the lymphocytes were positive for CD3 (Fig. 4b). Evaluation of other organs, including the liver, spleen, lungs, and bone marrow, revealed no histological evidence of infiltration. Based on these findings, we made a definite diagnosis of HTLV-1-related primary CNS lymphoma.

## Discussion and conclusions

We presented the case of a patient with relatively common LC along with depressive symptoms; however, the diagnosis was delayed due to anchoring bias and the patient’s work history before affliction. In general, primary CNS lymphoma due to adult T cell leukemia/lymphoma often presents as a mass [15]; however, LC, which is also a primary CNS lymphoma, does not present as a mass [4]. Currently, there is no established definition for diagnosing LC. However, studies have adhered to the description of gliomatosis cerebri in the criteria established by the 2007 World Health Organization classification of tumors of the CNS. These criteria include: (1) T2-weighted image/FLAIR abnormal hyperintensity present in at least three cerebral lobes or three anatomical regions of the CNS and (2) non-enhancing lesions or lesions without nodular or mass enhancement visible on initial MRI scans [7, 16]. In addition, excluding the presence of concomitant systemic lymphoma or intravascular lymphoma is crucial. In the present case, the diagnosis of LC was confirmed using imaging and pathological findings.

Symptoms of primary CNS lymphoma associated with adult T cell leukemia/lymphoma typically commonly observed include muscle weakness, altered mental state, paresthesia, headache, and urinary incontinence [17]. In contrast, cognitive deterioration and gait disturbances, as presented in the current case, are typical symptoms of LC and are less prevalent in primary CNS lymphoma with adult T cell leukemia/lymphoma [4]. Some patients present with depression as the main symptom [5], and the present patient’s clinical course was typical for LC. Two previous case reports of LC-like appearance on MRI due to adult T cell leukemia/lymphoma were identified [18, 19]. Table 1 presents the features of all three cases, including the present one. One case depicted the progression of lethargy, anorexia, and unsteadiness within approximately 1 month, while the other case illustrated the progression of headaches, mild dysarthria, and gait disturbances within approximately 2 months, mirroring the symptoms observed in the present case. These symptoms closely resemble those of LC [4]. Moreover, the diagnostic criteria for LC were satisfied based on the MR images [7, 16], and it is possible that these cases were LC. The case of “Taguchi, Sonobe, Yamato, Takeuchi, Ookawa, Kodama, Ohtsuki, and Miyoshi [19]” cannot be classified as a primary CNS lymphoma correctly, as T cells were also identified in the kidney and skin. In both cases, CSF analysis revealed almost normal cell counts, and a diagnosis of adult T cell leukemia/lymphoma was established through a brain biopsy. To date, no instances have been reported where a diagnosis was made subsequent to the detection of elevated cell counts in the CSF, as observed in the present case. In cases exhibiting indications of CSF involvement, the performance of CSF cytology can aid in establishing a definitive tissue diagnosis [12]. A comparison of MRI images of these cases showed brainstem involvement only in the present case.

**Table 1:** The features of all three cases, including the present one

Case	Age (year)	Sex	Chief complaint before admission	CSF leukocytes (cells/mm^3^)	Areas where infiltration was seen on MRI	Findings of biopsy	Time elapsed from symptom onset to mortality
Marshall 1998	42	Male	Four weeks history of progressive lethargy, anorexia, and unsteadiness	5−15	Right pons, midbrain, the internal capsule, the centrum semiovale, the substantia nigra, the globus pallidus, and the external capsule	A biopsy of the caudate nucleus showed a high-grade T cell lymphoma	3.5 months
Taguchi 1993	48	Female	Two months of headaches, mild dysarthria, and gait disturbances	2−3	The cerebral cortex to the subcortex, right putamen, pallidum, and cerebellum	A needle biopsy of the brain showed scattered infiltration of T cells in the dura and brain tissue An autopsy revealed that similar lesions were seen in the skin and kidney	8 months
Inaba 2023	49	Male	Three months of insomnia, anorexia, weight loss poor concentration, somnolence during the day, and gait disturbances	70	Both basal ganglia, thalamus, external capsule, brainstem, and temporal lobe, and the inferior aspect of the frontal lobe	An autopsy showed diffuse atypical lymphocytic infiltration and lytic necrosis throughout the brain tissue	4 months

In the present case, the CSF analysis performed on admission to the hospital revealed the presence of flower cells, which were a pivotal determinant in the diagnosis of LC. Notably, the diagnosis of LC through CSF cytology is rare. Although the incidence of abnormal CSF results in patients with LC is substantial, with a 14.3–85.7% ratio of abnormal-to-normal results, the presence of atypical lymphocytes has only been reported in a single case [7]. However, the presence of flower cells in the peripheral blood of individuals with adult T cell leukemia/lymphoma cells involves the CNS and can also be detected in the CSF, making the detection of flower cells in the CSF highly specific and aiding in narrowing down the diagnosis [20]. In the present case, the widespread progression of the CNS lesion at the time of admission might have led to the detection of flower cells in the CSF.

The diagnosis of LC was not made until approximately 3 months after symptom onset when the patient gradually developed impaired consciousness and gait disturbance and developed confusion. The importance of investigating organic causes in rapidly progressing psychiatric symptoms and dementia is often emphasized [21]. Therefore, it must be said that there was a diagnostic delay in this case. However, even in a 2016 systematic review, the median time from onset to diagnosis of LC is 4.5 months [4]. Generally, LC may be difficult to diagnose. This period may be shortened if LC is also added to the differential disease list at the onset of depressive symptoms and rapid progression of impaired consciousness and gait.

Furthermore, confusion developed rapidly, but the treatment for catatonia with generalized muscle tension was continued, which delayed diagnosis. According to the Diagnostic and Statistical Manual of Mental Disorders, Fifth Edition, diagnostic criteria for catatonia, three or more of the following symptoms are required: stupor, waxy flexibility, catalepsy, mutism, posturing, negativism, stereotypes, mannerisms, grimacing, agitation, echopraxia, and echolalia [22]. The patient met these criteria. Additionally, patients with catatonia and acute autonomic instability often require early therapeutic intervention with benzodiazepines or other drugs due to the increased risk of complications and death [23]. Therefore, prompt therapeutic intervention was necessary as the condition rapidly deteriorated, and a poor response to the administered treatment should have prompted the consideration of alternative diagnoses. However, the patient’s history of depressive symptoms following work-related changes likely anchored the diagnosis [24], making the consideration of alternative diagnoses difficult.

The most common lesions on MRI in LC are located in the deep and periventricular white matter, with the white matter areas of the frontal, parietal, occipital, and temporal lobes being the areas affected mainly [7]. This patient presented characteristic markedly symmetrical diffusion abnormalities in the bilateral internal capsule on DWI (Fig. 1). A previous case report of primary CNS lymphomas by natural killer/T cells demonstrated similar DWI findings, but the underlying cause was not determined, as no pathological autopsy was performed [25]. In the present case, a pathological autopsy was performed, which only showed diffuse atypical lymphocytic infiltration and lytic necrosis throughout the brain tissue. The cause of the characteristic imaging findings confined to the internal capsule in our patient is unknown. Since approximately 6 weeks had elapsed between MRI and autopsy, the lesion might have initially progressed along the pyramidal tract.

Additionally, upon admission, the case exhibited highly distinctive vital signs, as both systolic and diastolic blood pressure levels were elevated. Previous observational studies have revealed a consistent correlation between concurrent diastolic and systolic blood pressure levels and the presence of white matter lesions [26]. While a causal relationship between these lesions and elevated blood pressure remains uncertain, it is plausible that they might underlie hypertension in this particular case, given the absence of other contributing factors.

We believe that in instances of rapid progression of depression and cognitive decline, especially when the patient presents with a poor response to pharmacotherapy, head CT or MRI should be performed to exclude the possibility of LC prior to making a diagnosis of depression, even in patients with a typical history of depression. Further, we believe that LC needs to be further recognized as an important differential diagnosis for cognitive decline and depression.

## Data Availability

Data for this article are available upon request. Please contact the corresponding author for inquiries.

## Abbreviations

CNS: Central nervous system
LC: Lymphomatosis cerebri
HTLV-1: Human T cell leukemia virus type 1
CSF: Cerebrospinal fluid
MRI: Magnetic resonance imaging
